# Exploring the Binding Mechanism of PF-07321332 SARS-CoV-2 Protease Inhibitor through Molecular Dynamics and Binding Free Energy Simulations

**DOI:** 10.3390/ijms22179124

**Published:** 2021-08-24

**Authors:** Bilal Ahmad, Maria Batool, Qurat ul Ain, Moon Suk Kim, Sangdun Choi

**Affiliations:** 1 Department of Molecular Science and Technology, Ajou University, Suwon 16499, Korea; bilalpharma77@gmail.com (B.A.); mariabatool.28@gmail.com (M.B.); ainne.w@gmail.com (Q.u.A.); moonskim@ajou.ac.kr (M.S.K.); 2 S&K Therapeutics, Campus Plaza 418, Ajou University, Suwon 16502, Korea

**Keywords:** COVID-19, SARS-CoV-2, PF-07321332, α-ketoamide, 3CL protease, main protease

## Abstract

The novel coronavirus disease, caused by severe acute respiratory coronavirus 2 (SARS-CoV-2), rapidly spreading around the world, poses a major threat to the global public health. Herein, we demonstrated the binding mechanism of PF-07321332, α-ketoamide, lopinavir, and ritonavir to the coronavirus 3-chymotrypsin-like-protease (3CL^pro^) by means of docking and molecular dynamic (MD) simulations. The analysis of MD trajectories of 3CL^pro^ with PF-07321332, α-ketoamide, lopinavir, and ritonavir revealed that 3CL^pro^–PF-07321332 and 3CL^pro^–α-ketoamide complexes remained stable compared with 3CL^pro^–ritonavir and 3CL^pro^–lopinavir. Investigating the dynamic behavior of ligand–protein interaction, ligands PF-07321332 and α-ketoamide showed stronger bonding via making interactions with catalytic dyad residues His41–Cys145 of 3CL^pro^. Lopinavir and ritonavir were unable to disrupt the catalytic dyad, as illustrated by increased bond length during the MD simulation. To decipher the ligand binding mode and affinity, ligand interactions with SARS-CoV-2 proteases and binding energy were calculated. The binding energy of the bespoke antiviral PF-07321332 clinical candidate was two times higher than that of α-ketoamide and three times than that of lopinavir and ritonavir. Our study elucidated in detail the binding mechanism of the potent PF-07321332 to 3CL^pro^ along with the low potency of lopinavir and ritonavir due to weak binding affinity demonstrated by the binding energy data. This study will be helpful for the development and optimization of more specific compounds to combat coronavirus disease.

## 1. Introduction

The recent outbreak of coronavirus disease 2019 (COVID-19) caused by the novel severe acute respiratory syndrome coronavirus 2 (SARS-CoV-2) affected more than 192 million people and killed 4.1 million across the globe (as of 23 July 2021) [1,2]. The genome sequence of SARS-CoV-2 is closely related to SARS-CoV and β-coronavirus, sharing 79.5% and 96.2% sequence identity, respectively [3,4,5]. Coronaviruses belong to the Coronaviridae family and are found in birds and mammals [6]. The genomic organizations of SARS-CoV and MERS-CoV are similar [7] and both viruses comprise two polypeptides, pp1a and pp1ab. These polypeptides are processed into nonstructural proteins that play a fundamental part in the replication of these viruses, and the whole process is mediated by two kinds of main proteases: 3-chymotrypsin like protease (3CL^pro^) and papain-like proteases [8,9].

3CL^pro^, also called the main protease, consists of 306 amino acid residues, and is known to cleave at 11 sites in the polyproteins. The cleaving phenomenon leads to the formation of a helicase, single-stranded-RNA–binding protein, RNA-dependent RNA polymerase, 2′-*O*-ribose methyltransferase, endoribonuclease, and exoribonuclease [10,11]. 3CL^pro^ forms a homodimer, and each subunit consists of three domains. The substrate-binding site of 3CL^pro^ contains four subsites, S1′, S1, S2, and S4, and is highly conserved among all coronaviruses [3]. The active site of 3CL^pro^ is located in a cleft between domains I and II, while domain III helps with the formation of the dimer and is connected to domain II via a long loop (residues 184–199) [3,8]. A 3CL^pro^ antagonist will be highly specific to SARS-CoV-2 and will have minimal side effects because 3CL^pro^ shares no homology with human proteases [3,12]. Owing to the essential role of 3CL^pro^ in the transcription and replication of the viral genome and strong conservation of its binding-pocket residues, it is considered an ideal drug target in SARS-CoV-2 and other coronaviruses.

**Figure 1:**
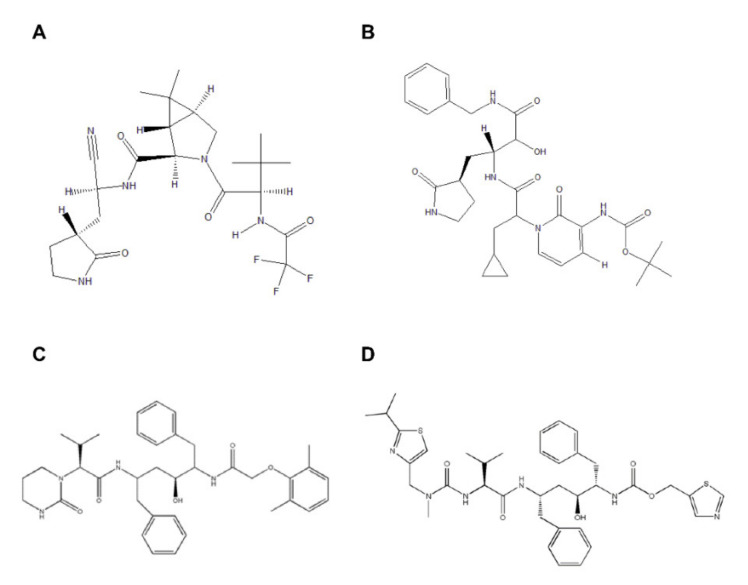
2D structure representation of (**A**) PF-07321332, (**B**) α-ketoamide, (**C**) lopinavir, and (**D**) ritonavir.

To date, more than 26% of world population has received at least one dose of COVID-19 vaccine [13]. The COVID-19 pandemic requires not only prevention via vaccine but also drug treatment. Antiretroviral drugs have been tested in past human coronavirus infections and against SARS-CoV-2, but a recent clinical trial of lopinavir and ritonavir failed to show any clinical benefit in COVID-19 disease. Protease inhibitors are used to treat HIV/AIDS and hepatitis C, but PF-07321332 (Figure 1A) is the first oral protease inhibitor to target the SARS-CoV-2 virus. In addition to PF-07321332, α-ketoamide (Figure 1B) has been reported to show in vitro inhibition of 3CL^pro^ [14]. The bespoke clinical candidate PF-07321332 (NCT04756531) is a potent protease inhibitor with potent antiviral activity against SARS-CoV-2.

The development of new drugs or vaccines is time-consuming; therefore, a drug-repurposing strategy was approved for the treatment of COVID-19 at this critical time [15]. The use of FDA-approved anti–HIV-I drugs lopinavir and ritonavir (Figure 1C,D) in combination against MERS-CoV and SARS-CoV has been reported earlier [16,17,18,19]. This combination of lopinavir and ritonavir is currently in a phase II trial along with interferon β-1b against MERS-CoV [16]. In contrast, for the treatment of MERS, this regimen is in phase IV of clinical trials in combination with Arbidol^®^ hydrochloride and oseltamivir (NCT04255017).

PF-07321332 is a 3CL^pro^ inhibitor with potent in vitro antiviral activity against SARS-CoV-2 and other coronaviruses (NCT04756531). Protease inhibitors interfere with the cutting step of the protease enzyme reaction. These drugs interact with the protease and block its polypeptide cutting ability. Lopinavir is used as a retroviral-protease inhibitor for the treatment of HIV-I infection and is usually co-administered with ritonavir, which increases the half-life of lopinavir and inactivates cytochrome P450 3A4 [20,21]. PF-07321332 is administered in combination with low doses of ritonavir as a booster to increase the bloodstream levels of PF-07321332. In the present study, the binding mechanisms of PF-07321332, α-ketoamide, lopinavir, and ritonavir were explored through molecular dynamics (MD) simulations and binding energy calculations. This study offers a deeper understanding of the mechanism of binding of these drugs to 3CL^pro^ and will be helpful to identify potent protease inhibitor candidates for SARS-CoV-2 and other coronaviruses. In future, our data can be used to identify novel drug candidates using supervised machine learning methods [22].

## 2. Results

### 2.1. Comparative Analysis of PF-07321332, α-ketoamide, Lopinavir, and Ritonavir Binding to 3CL^pro^

The crystal structure of 3CL^pro^ in complex with PF-07321332, lopinavir, and ritonavir is not yet available. Therefore, the docked complex of PF-07321332, lopinavir, and ritonavir with 3CL^pro^ was generated using the Molecular operating environment (MOE) software. Among the top 10 docking solutions, the best ligand conformation was selected based on the docking score and binding pose. In addition, the structure of α-ketoamide co-crystalized with 3CL^pro^ (PDB ID: 6Y2F) was retrieved from the RCSB PDB database.

**Figure 2:**
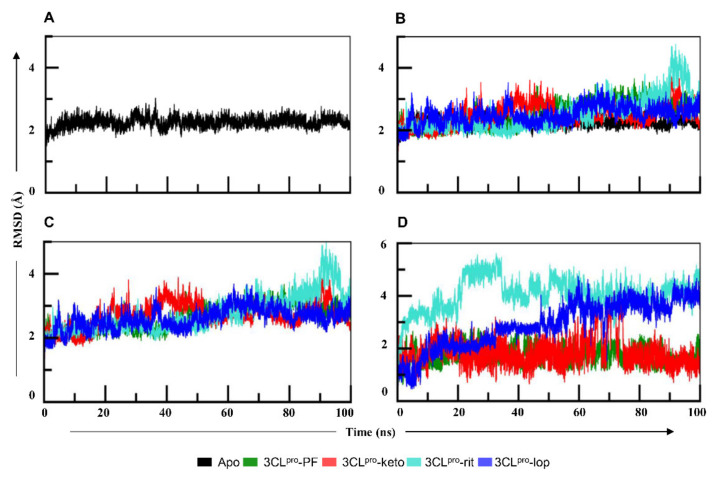
(**A**) The root mean square deviation (RMSD) graph of the apo form of 3CL^pro^ during the 100 ns simulation. The graph suggests that protein was stable during the whole simulation, with an average RMSD of 2.2 Å. (**B**) The RMSD graphs of all four proteins in complexes in comparison with the apo form. The protein in 3CL^pro^–PF and 3CL^pro^–keto remained stable whereas in 3CL^pro^–lop and 3CL^pro^–rit, it showed more fluctuations than in the other two 3CL^pro^ systems. (**C**) The RMSD graph of 3CL^pro^ complexes. The 3CL^pro^–rit and 3CL^pro^–lop complexes showed similar trend as the proteins. (**D**) RMSD graphs of the ligands in complexes with 3CL^pro^. The ligands PF-07321332 and α-ketoamide seemed stable, whereas both lopinavir and ritonavir in 3CL^pro^ complexes featured noticeable fluctuations. PF—PF-07321332; keto—α-ketoamide; rit—ritonavir; lop—lopinavir.

To elucidate the mechanism of binding of PF-07321332 (PF), α-ketoamide (keto), lopinavir (lop), and ritonavir (rit) with 3CL^pro^, all complexes were subjected to 100 ns simulations in Gromacs. To assess the stability of all four systems, root mean square deviation (RMSD) values were calculated for each ligand and protein individually and in complexed form, as presented in Figure 2. RMSD of apo-3CL^pro^ at 45 ns indicated a deviation up to 3 Å, after which the system was stabilized and oscillated in the mean range of 2.5 Å (Figure 2A). The protein in 3CL^pro^–PF (Figure 2B) remained stable throughout simulation with an average RMSD of 2.7 Å, while in 3CL^pro^–keto, the protein fluctuated in a 30–50 ns interval. The protein in 3CL^pro^–rit showed RMSD rising above 3 Å after 60 ns with higher fluctuation at 90 ns. The RMSD protein graph of 3CL^pro^–lop and 3CL^pro^–rit deviated from the apo-form. The 3CL^pro^–rit showed sinusoidal behavior until 60 ns and higher peak in the 60–80 ns time interval. Nevertheless, the RMSD of the protein in 3CL^pro^–lop increased gradually from 60 ns onwards (Figure 2B). It also manifested fewer fluctuations initially than apo-3CL^pro^ did until 40 ns, and then both systems yielded somewhat similar patterns up to 75 ns, and suddenly, the protein in 3CL^pro^–rit showed a remarkable deviation between 75 ns and 100 ns (Figure 2B). While assessing the RMSD of all four complexes (Figure 2C), we noticed that the 3CL^pro^–PF complex remained stable during the simulation. The RMSD of 3CL^pro^–keto complex fluctuated between 20–50 ns and then gradually stabilized. 3CL^pro^–rit and 3CL^pro^–lop complexes showed similar trend as their proteins. The RMSD graph of the ligands (Figure 2D) indicated that lopinavir and ritonavir featured greater deviations in comparison with other two ligands, whereas PF manifested the least deviations. The ligand in the 3CL^pro^–keto complex showed high jumps around 70 ns but quickly gained stability with a RMSD value of 1.5 Å. RMSD of the ligand in 3CL^pro^–lop showed increasing trend from 45 ns onwards. The ligand in 3CL^pro^–rit manifested a sudden increase in RMSD after 20 ns and fluctuated between 3 and 4.5 Å but attained stability later. We concluded that inhibitors PF and α-ketoamide imparted stability to the protein in their complexes.

**Figure 3:**
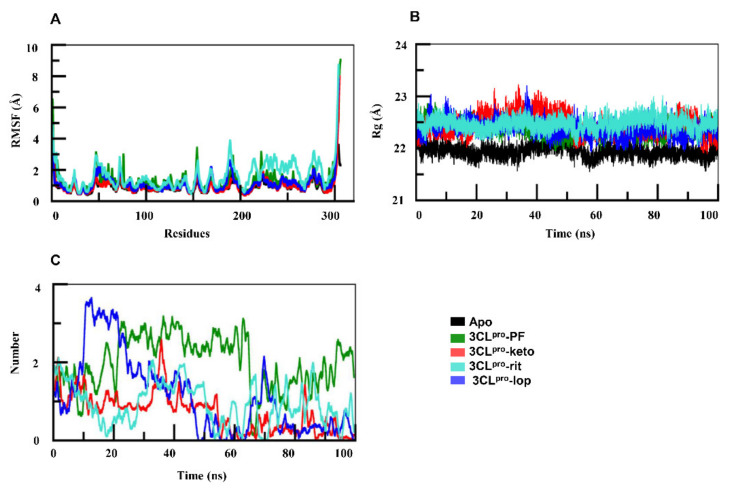
(**A**) Root mean square fluctuation graphs of apo-3CL^pro^ and of 3CL^pro^ complexes. In comparison with the apo-form, regions spanning amino acid residues 45–75 and 150–200 in both docked complexes showed deviations. (**B**) Radius of gyration of the 3CL^pro^ in complexes was higher than that of its apo-form. (**C**) Number of hydrogen bonds in the four complexes. PF—PF-07321332; keto—α-ketoamide; rit—ritonavir; lop—lopinavir.

To analyze the dynamic behavior of protein residues, root mean square fluctuation (RMSF) values were assessed. As compared with the apo form, RMSF (Figure 3A) of the protein in 3CL^pro^–rit indicated that amino acid residues 45–75 in the spanning region fluctuated by more than 3 Å. By contrast, in 3CL^pro^–lop, the residues of 3CL^pro^ between positions 45 and 75 fluctuated in the range similar to that of its apo form, i.e., 2.5 Å (Figure 3A). The rest of the protein residues of both complexes oscillated equally relative to the apoprotein. Overall, the residues of the 3CL^pro^ in complex with the ligands PF and α-ketoamide did not undergo any fluctuation, while in 3CL^pro^–rit complex, the protein featured the greatest fluctuations in comparison with the apo form.

To assess the compactness of protein structure, we calculated the radius of gyration (Rg). This parameter of the protein in 3CL^pro^–PF (Figure 3B) remained in the steady state and close to that of the apoprotein during the simulations, which means that the folding of the protein in 3CL^pro^–PF complex remained undisturbed. Figure 3B illustrates that the apoprotein followed a similar trend and oscillated at ~22 Å. The Rg of the protein in 3CL^pro^–keto fluctuated between 20–50 ns; thereafter, it retained steady state for the rest of the simulation. Rg of 3CL^pro^–rit remained steady up to 50 ns, then it gradually increased. 3CL^pro^–lop fluctuated with the highest peak of 23 Å near time point 39 ns, indicating that protein folding was disturbed. Lopinavir and ritonavir might have led to the unfolding in the 3CL^pro^ and thus exhibited increasing trend in Rg of the protein.

Next, the number of hydrogen bonds was calculated to evaluate the protein–ligand interactions during the simulations. The PF formed three hydrogen bonds (Figure 3C), and this remained consistent throughout the time trajectory. The α-ketoamide formed two bonds initially but their number dropped to one after 50 ns. The lopinavir initially formed three hydrogen bonds with 3CL^pro^, but their number gradually decreased, and the hydrogen bonds disappeared around 48 ns. Lopinavir formed two bonds in the beginning and ritonavir formed one, but these interactions were inconsistent throughout the MD simulations (Figure 3C). Therefore, the hydrogen bonds formed by lopinavir and ritonavir with 3CL^pro^ are inconsistent and weak as compared with the interactions of 3CL^pro^ with PF-07321332 and α-ketoamide.

### 2.2. Interaction Analysis of PF-07321332, α-ketoamide, Lopinavir, and Ritonavir with 3CL^pro^

**Figure 4:**
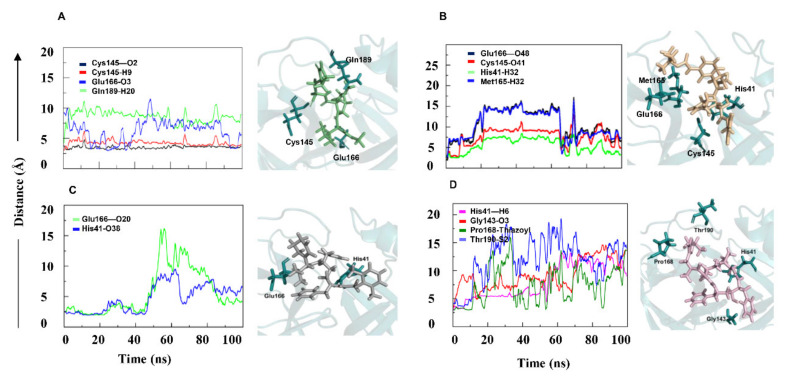
The minimum distance graph of protein–ligand interactions. The interaction distance between the protein and ligand in 3CLpro–PF (**A**) and 3CL^pro^–keto (**B**) remained constant. Interaction in 3CL^pro^–lop (**C**) initially remained constant but showed major fluctuations afterwards. In 3CL^pro^–rit (**D**), the lengths of all four bonds continuously increased. PF—PF-07321332; keto—α-ketoamide; rit—ritonavir; lop—lopinavir.

To evaluate the ligand–protein interactions, we computed the minimum distance between interacting atoms of the ligand and protein residues. Our analysis revealed the dynamics of the interaction of PF-07321332, α-ketoamide, lopinavir, and ritonavir with 3CL^pro^ (Figure 4). In 3CL^pro^–PF, three hydrogen bonds were formed (Table 1). The distance of Cys145–O2, Glu166–O3, and Gln189–H20 bonds remained within 4 Å (Figure 4A). The catalytic dyad residue Cys145 made strong bonds with atoms O2 and H9 as they remained intact during the simulation. This showed that the PF-07321332 has high affinity towards 3CL^pro^. The 4-methoxylindole group interacted through a hydrogen bond with the backbone of Glu166, a key residue located in the middle of the binding site. The study of Glu166 mutation, carried out in SARS-CoV 3CL protease (96% identity to SARS-CoV-2 3CL^pro^), has corroborated the role of this key residue in the substrate-induced dimerization [23]. The trifluoroacetamide moiety of the PF-07321332 compound involved in the Gln189–H20 bond accommodated the cyclopropyl-proline moiety to occupy the central portion of the binding site. Subsequently, the pyrrolidone moiety rearrangement led to bond formation of reactive nitrile group with Cys145. According to the analysis of the entire trajectory of 3CL^pro^–PF complex, PF-07321332 contacted Leu141 and Gln142. Leu141, Gln142, and Cys145 were part of the oxyanion loop (residues 138–145), which is lining the glutamine binding pocket and is presumably involved in the stabilization of the tetrahedral acyl transition state [24]. The ligand in 3CL^pro^–keto made three hydrogen bond interactions (Glu166–O48, Cys145–O41, and Met165–O48) and one arene interaction, His41–H32 (Figure 4B). The cyclopropyl moiety facilitated ligand binding in the shallow substrate-binding pocket at the surface of the 3CL^pro^. The α-keto group of the inhibitor interacted with Cys145. The study reported that thiohemiketal formation occurred in a reversible reaction by the nucleophilic attack of the catalytic Cys145 on the ligand [14]. In our study, α-ketoamide occupied the substrate binding space and its carbonyl oxygen formed hydrogen bond with the main-chain oxygen of Glu166. All these interactions kept fluctuating as the distance between the interacting entities continuously changed due to the bulky nature of the ligand. Similarly, in the 3CL^pro^–rit complex, three hydrogen bonds and an arene-type interaction were formed (Figure 4D). The length of His41–H6 and Gly143–O3 bonds remained within 7 Å during the initial 50 ns and continuously increased onwards. In the arene-type interaction, the distance between Pro168 and the thiazolyl group fluctuated within 5–10 Å during the simulation. The hydrogen bond Thr190–S2 appeared during the initial 10 ns, and the bond length was within 5 Å, but it diminished after 10 ns (Figure 4D). Two hydrogen bonds formed in 3CL^pro^–lop. Initially, the bond length of His41–O38 and Glu166–O20 (Table 1) was less than 5 Å, but after 50 ns, an increase in the bond length was detected because of ligand fluctuations, as depicted in the RMSD graph (Figure 2D and Figure 4C).

### 2.3. Binding Free Energy Calculation

Protein–ligand binding affinity is essential in understanding molecular recognition. The bespoke antiviral clinical candidate PF-07321332 (NCT04756531) is a potent 3CL^pro^ inhibitor with antiviral activity against SARS-CoV-2. The relative binding free energy of PF-07321332, α-ketoamide, lopinavir, and ritonavir with 3CL^pro^ was computed. To calculate the binding affinity of these ligands with 3CL^pro^, we extracted 1000 frames from the simulation trajectories. The g_mmpbsa (Table 2) computation revealed that the binding affinity of PF-07321332 for 3CL^pro^ was higher in comparison with α-ketoamide, lopinavir, and ritonavir. Relative to α-ketoamide, PF-07321332 showed more considerable binding energy toward 3CL^pro^, almost by 28 kJ/mol, and three times more than lopinavir and ritonavir. These data showed that PF-07321332 had a stronger binding affinity for 3CL^pro^.

## 3. Discussion

Combating the COVID-19 pandemic requires both prevention, including vaccine-based prevention, and targeted treatment for those who get infected. Given the way SARS-CoV-2 is evolving into new genetic variants and the worldwide impact of COVID-19, it is likely that access to treatment alternatives is crucial, now and beyond the pandemic. The frequent mutations aid the virus to infect different hosts and increase the virulence and transmissibility of SARS-CoV-2. The RNA viruses, including SARS-CoV-2, develop one mutation per replication cycle, which means RNA viruses may adapt to new environment, but these viruses are also restricted in their ability to change their genomes because they must keep their mutation rate low in order to survive [25]. Several mutations have been reported in the viral genome so far; 106 major amino acid substitutions were found to be responsible for increased virulence and transmissibility of the virus [26]. Most of these missense mutations occurred in structural proteins of SARS-CoV-2, such as spike (S) [27] and nucleocapsid (N) proteins. Only one point mutation, G15S, has been predicted using in silico studies in 3CL^pro^, but this residue is far from the active site and has a destabilizing effect on the protein structure [26]. Therefore, a virus comprising such mutations with a destabilizing effect would not last for long and would have reduced virulence. Only stabilizing mutations, such as in the S protein, increase the virulence and transmissibility of the virus [28]. A recent study has reported that the significant key mutations are located at the inverted repeat regions and CpG islands. These findings showed the role of mutations in the instability of the viral genome [29].

In this study, we have explored the binding of PF-07321332, α-ketoamide, lopinavir, and ritonavir to SARS-CoV-2 3CL^pro^ through MD simulation and MMPBSA calculation. PF-07321332 (NCT04756531) and α-ketoamide molecules that specifically bind to and inhibit SARS-CoV-2 3CL^pro^ could be promising alternatives to fight the pandemic [14]. The comparative binding mode analysis of PF-07321332, α-ketoamide, lopinavir, and ritonavir might provide a glimpse into designing rational drugs through the modification of the inhibitors based on the residues in the active site of the enzyme.

To determine the mechanism of binding of PF-07321332, α-ketoamide, lopinavir, and ritonavir to 3CL^pro^, molecular docking of these molecules was done. The best docking solutions for PF-07321332, lopinavir, and ritonavir were selected based on the docking score and the best binding pose of the ligand. To understand the binding mechanism of PF-07321332 and α-ketoamide along with the two antiretroviral drugs, lopinavir and ritonavir, to 3CL^pro^, MD simulations were performed for 100 ns. Backbone RMSDs of the 3CL^pro^–PF and 3CL^pro^–keto complexes were stable, whereas the 3CL^pro^–lop complex featured a deviation at 60–80 ns and the 3CL^pro^–rit complex a deviation between 75 ns and 100 ns (Figure 2B). RMSD deviation of the 3CL^pro^ protein was also analyzed in the ligand-bound form. The RMSD of 3CL^pro^ (Figure 2C) was found to be stabler in association with either PF-07321332 or α-ketoamide than with lopinavir or ritonavir. In contrast to the other systems, 3CL^pro^ in complex with ritonavir featured marked deviation between 88 and 95 ns of the simulation. Both ligands PF-07321332 and α-ketoamide are stabler with 3CL^pro^ in comparison with lopinavir and ritonavir because later two underwent marked deviation during the simulation (Figure 2D). The recent clinical trials have also showed these antiretrovirals are not efficacious as they did not significantly accelerate clinical improvements in serious COVID-19 patients [30]. Our binding energy data also showed low affinity of these drugs towards 3CL^pro^. To assess the impact of ligand binding on protein residues, RMSF of 3CL^pro^ were analyzed in all four complexes. With either lopinavir or ritonavir, amino acid residues 45–65 and 145–200 showed greater fluctuations in comparison with the apo-form (Figure 3A). These fluctuating regions mainly consist of binding-site residues that are interconnected by a hydrogen bond network and are involved in catalytic dyad formation between His41 and Cys145 [31,32,33]. However, the RMSF of the protein in 3CL^pro^–PF showed similar trend as its apo-form. Compactness, which determines stability of a protein, was evaluated for 3CL^pro^ using Rg. The compactness of 3CL^pro^ in complex with PF-07321332 and α-ketoamide remained in steady state the same as the one of its apo-form (Figure 3B). Rg of 3CL^pro^ in its complex with ritonavir or lopinavir was higher than that of apo-3CL^pro^. Nonetheless, 3CL^pro^ compactness after PF-07321332 and α-ketoamide binding remained constant throughout the simulation.

One of the most interesting examples of MD application is the drug discovery area, where this method drives experiments [34,35]. To analyze the interactions of the four molecules with 3CL^pro^, the minimum distance between the atoms of interacting residues of each protein and ligand (PF-07321332, α-ketoamide, lopinavir, and ritonavir) were calculated as a function of simulation time to analyze the dynamic behavior of the interacting groups. In the interaction analysis, PF-07321332 and α-ketoamide showed stronger interactions with 3CL^pro^, and these interactions remained intact during the simulation because the minimum distance between the interacting groups stayed almost unchanged. The interactions, i.e., Cys145–O2 and Cys145–H9 in 3CL^pro^–PF (Figure 4A), remained intact during the simulation. This showed that PF-07321332 was strongly bonded to 3CL^pro^ and capable to disrupt the His41–Cys145 catalytic dyad, which, together with the N-terminus residues 1 to 7, is thought to have a vital role in proteolytic activity [36]. The Glu166 anchor hooks the ligand tightly to the central region of the binding site, which facilitate the formation of further interactions with other residues. Gly143 of SARS-CoV-2 3CL^pro^ was reported to be the most favorable residue to form hydrogen bonds with ligands, followed by Glu166, Cys145, and His163 [37]. The amides of Gly143, Cys145, and Ser144 form the cysteine protease’s canonical “oxyanion hole”. The interaction of PF-07321332 and α-ketoamide with the catalytic site residues may cause the distortion of the oxyanion hole in the reaction mechanism, and it may lead to the inhibition of 3CL^pro^ in SARS-CoV-2. Compared with PF-07321332 and α-ketoamide, both antiretroviral drugs lopinavir and ritonavir manifested weaker interactions with 3CL^pro^, judging by binding energy values (Table 2). PF-07321335 interacts with Cys145, thereby disrupting His41–Cys145 catalytic dyad. The α-ketoamide has the same mechanism as reported earlier carries; a nucleophilic attack of the catalytic Cys145 on the α-keto group of the inhibitor, disrupting the His41–Cys145 catalytic dyad [14]. Our binding energy data (Table 2) revealed that PF-07321335 has higher affinity towards 3CL^pro^. This is in accordance with PF-07321335 showing a potent inhibition of 3CL^pro^ (NCT04756531). Lopinavir and ritonavir showed weaker interactions because only two residues, His41 and Glu166, were found to be involved in hydrogen bonding. The minimum distance of 5 Å between these interacting entities persisted up to 45 ns; after that, it fluctuated. In contrast with PF-07321335 and α-ketoamide, lopinavir and ritonavir engaged in interactions with 3CL^pro^, but these interactions varied substantially. Some chemical substitutions in lopinavir and ritonavir (such as the addition of an α-keto group) can act as electrophiles that may prevent the His41–Cys145 dyad formation by a nucleophilic attack of Cys145. Although in our simulations, lopinavir and ritonavir engaged in interactions with His41 and Cys145 along with other catalytic site residues, these contacts fluctuated considerably, as evident from our interaction analysis. Coronavirus protease 3CL^pro^ lacks a C2-symmetric pocket, through which lopinavir and ritonavir bind to HIV-I protease. The absence of a C2-symmetric pocket in coronavirus 3CL^pro^ could be the reason why these drugs do not bind to 3CL^pro^, which makes them ineffective in COVID-19 patients. The binding energy data (Table 2) showed that lopinavir and ritonavir have weak affinity to 3CL^pro^.

Conclusively, 3CL^pro^ is an important drug target, which cleaves the viral polyprotein at 11 different cleavage sites and is required for viral maturation. The druggability of 3CL^pro^ has already been demonstrated in various studies. The mutations in the dominant variants of SARS-CoV-2, such as B.1.1.7 and B.1.617, with higher transmissibility rate were studied and it has been demonstrated that most of the mutations have been found in the receptor binding domains or structural proteins, but no mutation has been reported in 3CL^pro^ so far [38]. Therefore, 3CL^pro^ is an attractive target for anti-COVID-19 drug discovery. In recent studies, many new compounds have been reported as potential inhibitors of 3CL^pro^ [14,39,40,41,42]. We included four of the potential inhibitors in our study, compared their binding energy and discussed their interactions with the protein to identify the potent inhibitor. We found that the compound PF-07321332, currently in clinical trials, was the best of these four compounds. In addition to these molecules, sterenin M was also reported in a recent computational study [39] to bind to the same active binding site as PF-07321332 and interact with the residues of catalytic dyad, but its total binding energy profile showed that the binding of PF-07321332 with protein was much stabler than the one of sterenin M [39]. In similar studies, the identified compounds bind to the same binding site, but the stability of complex is lower than the one of PF-07321332 [40,41]. Hence, considering the current pandemic situation, it is crucial to find some potent drug candidate with good binding affinity. The MD simulation and MMPBSA are widely used for assessing protein–ligand binding affinity, but there are a few limitations of the MD simulations, such as many biochemical processes including receptor conformational shifts relevant to drug binding occur on time scales that are much longer than those amenable to simulations. In MD simulations, each atom is assigned a fixed partial charge before simulation. However, the electron clouds in surrounding atoms shift continuously according to their environments. A dynamic and responsive representation of atomic partial charges would be more accurate.

## 4. Conclusions

A detailed analysis of interactions of proteins and ligands represents an optimized method for the rational design of new compounds. Binding energy helps to identify a lead compound. MD, free energy perturbation, meta-dynamics, and other methods are consistently used for studying drug–target binding. Assessment of these methods may help to achieve the most definite target-optimized affinity with improved drug efficacy [43]. Integrating MD simulation with binding-free-energy calculation by means of g_mmpbsa to determine interaction free energy between a ligand and protein is an efficient method for distinguishing between active and inactive molecules [44]. The comparative analysis of PF-07321332, α-ketoamide, lopinavir, and ritonavir via MD simulation provided a detailed insight into the interactions of these compounds with 3CL^pro^. We believe that our findings revealed the binding mechanism of PF-07321332 and α-ketoamide and further explained the inability of lopinavir and ritonavir to cause clinical improvement in severe COVID-19 patients by due to their low affinity towards 3CL^pro^. We believe the dynamic interaction and binding energy of PF-07321332 and α-ketoamide will be helpful in designing potent inhibitors for 3CL^pro^.

## 5. Materials and Methods

### 5.1. System Preparation for Molecular Docking and MD Simulations

The crystal structure of SARS-CoV-2 3CL^pro^ (Protein Data Bank [PDB] ID: 6M03) and structures of 3CL^pro^ in complex with 3CL^pro^ (PDB ID 6Y2K) [45,46] were downloaded from the PDB [47]. The 2-D structure of PF-07321335 was drawn in Chemdraw and the three-dimensional (3D) coordinates of lopinavir (PubChem CID: 92727) and ritonavir (PubChem CID: 392622) were retrieved from the PubChem database [48] for molecular docking. All hydrogens and missing atoms were added to the proteins, while crystal water was removed. The MOE software from Chemical Computing Group Canada (Montreal, QC, Canada) [49] was used for energy minimization of 3CL^pro^ and both drugs to remove any steric clashes and to improve the bond lengths and bond angles. The default parameters were used for energy minimization, while the gradient was set to 0.01 rms kcal/mol.

### 5.2. Molecular Docking of 3CL^pro^ to Lopinavir and Ritonavir

PF-07321335, α-ketoamide, lopinavir, and ritonavir were docked into the binding pocket of 3CL^pro^ using the MOE software [49]. The key residues involved in ligand binding reported in the literature were selected for docking: His41, Asn142, Cys145, His164, Met165, Glu166, Gln189, and Thr190. The triangle matcher placement method was selected with the London dG scoring function, and the induced-fit method was applied for refinement of the binding poses of ligands. A total of 100 conformations were generated for each ligand, and the best pose with the lowest binding energy was selected. Complexes of 3CL^pro^ with PF-07321335, α-ketoamide, lopinavir, and ritonavir were analyzed and saved for MD simulations.

### 5.3. Building the Ligand Topology

Prior to MD simulations, the ligand topology was generated on the CHARMM general force field (CGenFF) server (University of Maryland, Baltimore, MD, USA) [50]. Ligands, along with their 3D coordinates, were extracted from their respective complexes and saved in mol2 file format, and all hydrogens were added. The mol2 file was uploaded to the CGenFF server, which generated a CHARMM-compatible stream file comprising ligand information, such as atom types, charges, and bond parameters. A Python script was executed to convert the stream files into Gromacs-compatible [51,52] files. These files were used for the MD simulations.

### 5.4. MD Simulations of Complexes

The dataset for MD simulations contained apo-3CL^pro^ and four 3CL^pro^ complexes. All five MD simulations were carried out using the Gromacs (University of Groningen, Groningen, Netherlands) software for 100 ns each, and the CHARMM36 forcefield [53] was applied to the systems. Periodic boundary conditions were used, and the protein was placed inside a 10 Å cubic box. For solvation of the system, the TIP3P water model [54] was used, and an appropriate amount of counterions was added to neutralize the system. The solvated electroneutral system was subjected to energy minimization by the steepest-descent algorithm, followed by temperature and pressure equilibration steps. The production run was carried out for 100 ns in each system, and data analysis was performed by means of Gromacs built-in tools, MOE, and PyMOL (Schrödinger, LLC. DeLano Scientific, San Francisco, CA, USA) [55,56].

### 5.5. Binding-Free-Energy Calculation

The molecular mechanics Poisson–Boltzmann surface area (MMPBSA) method was used to compute the binding free energy of all complexes. A thousand frames were extracted from each 100 ns simulation trajectory by means of the *gmx trjconv* utility of Gromacs. The extracted frames were used for free-binding-energy calculation with the help of the g_mmpbsa tool [44]. The total binding free energy (ΔG_binding_) was calculated as

ΔG_binding_ = ΔG_complex_ − (ΔG_protein_ + ΔG_ligand_)

where G_complex_ is the average free energy of the complex, and G_protein_ and G_ligand_ are the average energy values of the protein and ligand.

## Figures and Tables

**Table 1:** Residue and ligand atom interactions with the bond type and energy in 3CL^pro^ complexes.

Complex	Residue	Ligand Atom	Bond Type	Energy
3CL^pro^–PF	Cys145	O2	Hydrogen	−5.04
	Cys145	H9	Hydrogen	−5.04
	Glu166	O3	Hydrogen	−4.41
	Gln189	H20	Hydrogen	−3.01
3CL^pro^–keto	Glu166	O48	Hydrogen	−0.70
	Cys145	O41	Hydrogen	−0.80
	Met165	O48	Hydrogen	−0.80
	His41	H32	Arene	−0.90
3CL^pro^–lop	His41	O38	Hydrogen	−4.1
	Glu166	O20	Hydrogen	−1.6
3CL^pro^–rit	His41	H6	Arene	−3.0
	Gly143	O3	Hydrogen	−2.2
	Pro168	Thiozyl group	Arene	−0.8
	Thr190	S2	Hydrogen	−0.8

**Table 2:** Binding energy of 3CL^pro^ and HIV-I protease complexes as computed via the g_mmpbsa method.

Complex	VdW Energy	Electrostatic	Polar Solvation	SASA	Total Binding Energy (kJ/mol)
3CL^pro^–PF	−135.294 +/− 16.258	−39.293 +/− 25.780	96.026+/− 21.556	−23.441 +/− 2.526	−102.002 +/− 21.336
3CL^pro^–keto	−76.478+/− 25.052	−40.348 +/− 30.471	50.648 +/− 49.365	−7.887 +/− 3.036	−74.065 +/− 22.579
3CL^pro^–lop	−110.529 +/− 28.28	−65.740 +/−26.09	168.011 +/− 33.31	−25.239 +/−2.40	−33.497 +/− 26.52
3CL^pro^–rit	−75.994 +/− 60.285	−55.115 +/− 23.09	91.803 +/− 18.798	−4.219 +/− 8.798	−43.525 +/− 29.171

## Data Availability

The structure models and simulation trajectories are available upon request (sangdunchoi@ajou.ac.kr). All remaining data are contained within the manuscript.
